# Th17 Cells, Glucocorticoid Resistance, and Depression

**DOI:** 10.3390/cells12232749

**Published:** 2023-11-30

**Authors:** Julia N. Khantakova, Anastasia Mutovina, Kseniya A. Ayriyants, Natalia P. Bondar

**Affiliations:** 1 Institute of Cytology and Genetics, Siberian Branch of Russian Academy of Sciences (SB RAS), Prospekt Lavrentyeva 10, Novosibirsk 630090, Russia; kaayriyants@bionet.nsc.ru (K.A.A.); nbondar@bionet.nsc.ru (N.P.B.); 2 Department of Natural Sciences, Novosibirsk State University, Pirogova Street 2, Novosibirsk 630090, Russia; a.mutovina@g.nsu.ru

**Keywords:** depression, neuroinflammation, glucocorticoid resistance, interleukin-17, T-helper 17 lymphocites, gut–brain axis

## Abstract

Depression is a severe mental disorder that disrupts mood and social behavior and is one of the most common neuropsychological symptoms of other somatic diseases. During the study of the disease, a number of theories were put forward (monoamine, inflammatory, vascular theories, etc.), but none of those theories fully explain the pathogenesis of the disease. Steroid resistance is a characteristic feature of depression and can affect not only brain cells but also immune cells. T-helper cells 17 type (Th17) are known for their resistance to the inhibitory effects of glucocorticoids. Unlike the inhibitory effect on other subpopulations of T-helper cells, glucocorticoids can enhance the differentiation of Th17 lymphocytes, their migration to the inflammation, and the production of IL-17A, IL-21, and IL-23 in GC-resistant disease. According to the latest data, in depression, especially the treatment-resistant type, the number of Th17 cells in the blood and the production of IL-17A is increased, which correlates with the severity of the disease. However, there is still a significant gap in knowledge regarding the exact mechanisms by which Th17 cells can influence neuroinflammation in depression. In this review, we discuss the mutual effect of glucocorticoid resistance and Th17 lymphocytes on the pathogenesis of depression.

## 1. Introduction

Depression has a high degree of phenotypic and etiological heterogeneity and can be either an independent disease or a comorbid one. In a large umbrella review [1], the authors analyzed 17 systematic reviews and meta-analyses on the major strands of research on serotonin and found no conclusive evidence linking the pathogenesis of depression with changes in serotonin levels, and diagnostic methods for reducing serotonin availability using tryptophan depletion did not always cause depressive mood in the volunteers. The authors conclude that the huge research effort based on the serotonin hypothesis does not provide strong evidence for the biochemical basis of depression. All of this suggests that the available pharmacological drugs, which mainly affect the level of serotonin in the synaptic gap, are aimed at reducing the manifestations of depression, but do not eliminate the cause of the disease. The continued dominance of the serotonin theory provokes chronic drug abuse, causing a “fear of withdrawal” in patients and the accumulation of adverse effects on other organs. 

Depression is a complex disorder whose pathogenesis is based on changes in several systems that can mutually reinforce each other. In addition to serotonin, chronic stress, hormonal disorders, chronic inflammation, changes in the microbiome, etc., are also implicated in the development of depression, and can mutually affect each other and increase the negative impact on the pathogenesis of depression. Recent studies have also identified the role of IL-17A and more pathogenic and inflammatory T-helper cell 17 type (Th17) subpopulations in depression, especially treatment-resistant depression [2,3]. The Th17 lymphocyte is a unique T-helper cell line that differs from the classical Th1and Th2 cells by a set of synthesized cytokines, such as IL-17 (A-F), IL-21, and IL-22 [4,5,6], and is also known for its resistance to glucocorticoids (GCs) [4,7,8,9]. Despite recent advances, there is still a significant knowledge gap regarding the exact mechanism by which Th17 cells can influence neuroinflammation in depression. In this review, we want to discuss the effects of glucocorticoid resistance, inflammation, and the role that Th17 lymphocytes can play in the interaction of these factors in the development of depression.

### Role of Inflammation and Glucocorticoid Resistance in the Pathogenesis of Depression

Glucocorticoid resistance, hypersecretion of cortisol, and increased inflammation co-exist in depression. Treatment-resistant depression patients showed a simultaneous increase in cortisol and IL-6 levels [10], as well as a high correlation between a decrease in the expression of the glucocorticoid receptor (GRa) and the upregulation of the inflammatory genes in monocytes [11]. Moreover, in vitro peripheral blood mononuclear cells (PBMCs) of depressed patients respond poorly to dexamethasone and cortisol when stimulated with lipopolysaccharide [10]. As early as 1991, dexamethasone was shown not to inhibit mitogen-induced lymphocyte proliferation and IL-1b production in depressed patients, unlike in healthy donors [12]. Subsequent studies have shown that even healthy donors’ lymphocytes differ in their sensitivity to steroids [13].

Normally, under the influence of stress, corticotropin-releasing hormone (CRH) is released in the hypothalamus, which acts on the pituitary gland and stimulates the production of adrenocorticotropin (ACTH), which in turn affects the adrenal glands and causes the release of cortisol. An increase in cortisol in the blood triggers a loop of negative regulation, limiting the further release of CRH and ACTH. In chronic stress, the negative feedback loop is disrupted, which leads to a persistent increase in GR in the blood. Such abnormalities in hypothalamic–pituitary–adrenal (HPA) axis regulation and elevated blood cortisol levels are observed in patients with depression [14,15]. A similar disorder is also observed in Cushing’s syndrome, but this syndrome does not appear in patients with depression. Such changes in HPA regulation occur due to the development of glucocorticoid resistance, which may be associated with impaired function of GR, changes in the level of GR expression, changes in the bioavailability of GR, as well as the influence of the immune system on HPA [16,17].

Depression-related increases in pro-inflammatory cytokines, such as IL-6 and TNF [18,19,20,21], can, on one hand, further stimulate HPA and glucocorticoid release [22,23]. On the other hand, it is known that the pro-inflammatory cytokine IL-1 suppresses GR activation and translocation from the cytoplasm to the nucleus, thereby reducing the expression of GC-sensitive genes, forming glucocorticoid resistance [24]. In addition, cytokines can affect serotonin metabolism, reducing its synthesis and increasing reuptake [25]. They can also deplete neurotrophic factors such as BDNF, which play a neuroprotective role in depression [26]. Cytokines activate cellular cascades that cause excitotoxicity [27,28] and apoptosis, as well as inhibit neurogenesis in the hippocampus [29,30,31].

Thus, glucocorticoids and pro-inflammatory cytokines interact closely: increased production of pro-inflammatory cytokines and/or cortisol in the blood can both directly cause depression, and can also cause hyperactivation of the HPA axis, increase the resistance of glucocorticoid receptors, increase cortisol levels and cause the pro-inflammatory status of the immune system, forming a pathological vicious circle. We want to emphasize that the facts described above (glucocorticoid resistance and the level of pro-inflammatory cytokines) are not always coincident in depression, and are not characteristic for all patients. This further highlights the complexity of the pathogenesis of the disease and the need for further search for important links in the development of depression. 

## 2. Th17 Immune Cells and Neuroinflammation

Before starting to describe the possible pathogenetic effect of Th17 lymphocytes on the development of depression, we will first focus on the main points of development and functioning of this type of cell. A large amount of data on the biology of Th17 lymphocytes and their involvement in neuroinflammation was obtained by studying the experimental autoimmune encephalomyelitis (EAE), a model of multiple sclerosis (MS) in humans.

In 2003, it was shown for the first time that in EAE, the central nervous system (CNS) has an expansion of an IL-17-producing T-cell population [32] capable of developing autoimmunity during adaptive transfer [33], and IL-23 is necessary for stabilizing their functions [34]. These cells were named Th17 and were recognized as a unique subset of T-helper cells, characterized by the expression of RAR-related orphan receptor γ (RORγt) as master regulator [35], the production of inflammatory cytokines IL-17 (A-F), and also IL-21 and IL-22 (Table 1) [36,37]. Intensive study of the cells allowed us to discover the main differentiation factors necessary for the polarization of naïve T cells into Th17 lymphocytes. It has been shown that the synergistic effect of IL-6 and TGF-β in differentiating cells increases the synthesis and secretion of IL-21, which leads to an autocrine increase in the expression of the IL-23 receptor and interleukin-1 receptor antagonist protein (IL-1RN, formerly IL-1RA) [5,38]. Interestingly, TGF-β was initially considered an anti-inflammatory cytokine due to its positive effect on the differentiation of FOXP3-expressing regulatory T cells (Tregs) and its ability to block the differentiation of Th1 and Th2 lymphocytes [39]. Under physiological conditions, Th17 lymphocytes are found in the mucosal and epithelial barriers, such as the intestines, lungs, and skin, participating in maintaining homeostasis and protecting against extracellular bacterial and fungal pathogens, and are practically not found in the organ stroma [40]. On the other hand, their cytokine profile and ability to attract other types of immune cells allow them to enter organ stroma, where they are involved in the development of autoimmune inflammation, as shown in multiple disease models [41,42,43]. 

A detailed study of the biology of Th17 lymphocytes revealed the presence of functionally different cell sets that differ in their ability to participate in the triggering of autoimmune inflammation [44,45]. When culturing mouse naïve CD4+ T cells in vitro, the combination of IL-6 and TGF-β promotes the formation of non-pathogenic Th17 cells. Such cells are characterized by increased expression of the immunoregulatory genes *Il10*, *Il9*, *Maf*, and *Ahr*; produce cytokine IL-17 and high doses of the anti-inflammatory cytokine IL-10; and have a weak ability to trigger autoimmunity. The main role of non-pathogenic Th17 lymphocytes is to maintain the homeostasis of immune responses in barrier tissues, especially mucous membranes, including through the activation of innate immune response cells such as neutrophils and monocytes [46]. Under pro-inflammatory conditions, in the presence of IL-23, IL-1b, IL-6, IL-12, and TNF, highly pathogenic Th17 cells are developed [6,47] with increased expression of pro-inflammatory gene products, including *Csf2*, *Ifng*, *Tbx21IL23r*, and *Gzmb* [6,48]. IL-23R-signaling has been shown to be a key factor in the conversion of homeostatic Th17 cells to pathogenic ones [42]. IL-1b suppresses IL-10 production in Th17 lymphocytes and is also involved in their transformation into pathogenic cells [49]. In 2022, Thakore et al. performed combined ATAC-seq and RNA-seq, which revealed significant differences in the chromatin landscape of non-pathogenic and pathogenic Th17 lymphocytes [50]. Thus, the authors showed that the differentiation of pathogenic Th17cells is regulated by transcription factors such as Atf3, Bhlhe40, Fos, Nr4a1, Eomes and Tbx, Nr4a1, Eomes, and Tbx21, while for non-pathogenic Th17 cells, it is transcription factors RORγtγt, Ets 1, Batch 2, Fosl2, and Rbpj that are more important [50].

In addition, Th17 lymphocytes can exhibit cell plasticity [51] and, depending on the microenvironment conditions, can express various transcription factors and cytokines, demonstrating some properties of other subpopulations of Th lymphocytes [6,36,39,47]. In particular, the study of EAE showed that some pathogenic Th17 lymphocytes can reduce IL-17 expression, trans-differentiating into highly pathogenic IL-17A-IFNy+GM-CSF+ exTh17 lymphocytes producing IFN-γ and GM-CSF by increasing inflammation through additional involvement of innate immune system cells [40,42,52]. In addition, it has been shown that about 80% of CD4 + T cells infiltrating the central nervous system are Th17, and ~40% of these cells express IFN-y and ~40% express GM-CSF, indicating that a significant proportion of GM-CSF + T cells during EAE originate from Th17 lymphocytes [53]. However, blocking GM-CSF or IFN-y in vivo has little effect on the course of EAE in mice, whereas blocking IL-17 prevents its development [54]. Thus, although some Th17 lymphocytes may stop producing IL-17, IL-17 still has a pathogenic role in certain autoimmune diseases, either as an effector cytokine or in the priming of Th17 cells [54]. 

Th17 lymphocytes play an important role in tissue homeostasis, especially in barrier tissues, attracting monocytes and neutrophils to the focus of inflammation [40]. In patients with depression, leukocytosis is observed in the blood due to an increase in the number of neutrophils [55], and glucocorticoids can increase the activity of Th17 lymphocytes and their interaction with neutrophils [56]. Under the influence of IFN I type (IFN-α and IFN-β), an increase in which can induce the development of depression [57,58], neutrophils show a significant chemotactic effect on Th17 lymphocytes [46], with Th17 lymphocytes beginning to express higher levels of IL-23R and IL-17 mRNA [59,60], characteristic of pathogenic Th17 lymphocytes. 

To date, six cytokines of the IL-17 family (IL-17A–IL-17F) and five members of the IL-17 receptor family (IL-17RA–IL-17RE) have been identified [61]. IL-17A and IL-17F have the greatest homology and can form homo- (IL-17A/A and IL-17F/F) and heterodimers (IL-17A/F) [62]. Functionally, IL-17A, -C, and -F are pro-inflammatory cytokines [63]; conversely, IL-17E (previously IL-25) plays an anti-inflammatory role by participating in the induction of the Th2 immune response, further suppressing Th17 cells via the down-regulation of IL-23 [64]. IL-17B and IL-17D are the least studied cytokines in the family. IL-17 family receptors can form homo-and heterodimers and are expressed by different cell types. IL-17RA, the most expressed form of the receptor, can form homo- (IL-17RA/RA) and heterodimer complexes (IL-17RA/RC; IL-17RA/RD) and binds IL-17A, -C, -E, and -F [61,65]. While IL-17RA is expressed everywhere, IL-17RC expression is characteristic of non-hematopoietic epithelial and mesenchymal cells [66]. Recently, it has been shown that IL-17RD can serve as an alternative receptor subunit for IL-17A, but not for the homologous IL-17F [67]. The response pattern to IL-17 depends on the receptor complex and the characteristics of the cells expressing them. 

For the brain, IL-17A has been shown to induce glial cell activation and is involved in the initiation and development of CNS diseases, such as autoimmunity, autism, and stroke [68,69]. IL-17A disrupts the permeability of the blood–brain barrier (BBB), reducing the expression of the cerebral tight junctions, such as occludin and claudin-5, and increasing the number of adhesive molecules, such as chemokines and ICAM-1, facilitating access to the central nervous system and additionally increasing the transmigration of lymphocytes [70,71,72,73]. Under its influence, the expression of IL-17R and chemokines on the surface of endothelial cells increases [74], which contributes to the additional recruitment of Th17 lymphocytes to the central nervous system, regardless of the violation of BBB permeability [52]. In the presence of IL-17, as well as under the influence of IL-23 or IL-1b, microglia enhances the production of pro-inflammatory factors such as IL-6, macrophage inflammatory protein-2, nitric oxide, adhesion molecules, and neurotrophic factors [70], as well as IL-17 itself, thereby enhancing the effect of cytokine on the BBB and additionally attracting immune cells [71,75]. IL-17a is also able to reduce the absorption and transformation of glutamate by astrocytes, increasing the excitotoxicity of glutamate [76]. IL-17A knockout mice show a decrease in the level of pro-inflammatory cytokines in the dental gyrus, an increase in the number of postsynaptic AMPARs, and, probably, an increase in presynaptic glutamate release [29]. Mouse models have shown that even under physiological conditions endogenous IL-17A suppresses neurogenesis in the dentate gyrus of the hippocampus, by suppressing intrinsic neuronal excitability and decreasing the expression of proneuronal genes in neuronal progenitor cells [29].

It is worth noting that other cells are also capable of producing cytokines of the IL-17 family [77]. To date, CD8+ T cells, γδ T cells, innate lymphoid cells 3 type, natural killer (NK) cells, invariant NK T cells, mucosal-associated invariant T cells, mast cells, and neutrophils are known to be capable of synthesizing IL-17 [77], and are, in fact, the earliest responders in the immune response of barrier tissues with an evolutionarily programmed defense mechanism [78]. This excess of IL-17 production indicates the irreplaceability of the cytokine in immune responses and its initiating role in the initial stages of the immune response, which makes it possible to increase the cytokine level even before recruiting adaptive Th17 lymphocytes to the inflammation site. 

Studies with human cells have also identified subpopulations of Th17 lymphocytes that are transcriptionally similar to in vitro generated mouse non-pathogenic and pathogenic Th17 lymphocytes [19,79,80]. Taking into account that the main reservoir of homeostatic Th17 lymphocytes is the intestinal mucosa [42,44], it becomes obvious that the state of the microbiome will have a significant impact on the effector properties of Th17 lymphocytes. 

### Gut–Brain Axis and Th17 Immune Response

Studies in animal models have shown that stress affects not only the development of anxiety and depressive behavior but also changes the composition of the microflora and the pro-inflammatory status of the immune system [81,82,83]. In animals with depressive-like behavior, the number of Th17 lymphocytes and the Th17/Treg ratio increased in the spleen, liver, and ileum [81]. Among patients with clinically significant symptoms of depression, an altered microbiota composition was also found [84,85]. For example, a negative correlation is shown between a decrease in short-chain fatty acid-producing species (e.g., *Faecalibacterium prausnitzii* and *Clostridium leptum*) [85] and the severity of depression [86]. Both bacterial species are known to maintain Th17Th/Treg balance to promote Foxp 3-Treg differentiation and block the IL-6/STAT3/IL-17 downstream pathway and Th17 cell differentiation [87,88].

The intestinal tract contains most of the body’s lymphatic tissue. Most T lymphocytes in the intestinal epithelium and lamina propria have an effector phenotype and are believed to be involved in maintaining a homeostatic balance between the luminal commensal microflora and intestinal tissues. Due to the large presence of Th17 lymphocytes in the intestinal wall, it has become obvious that the intestinal microflora can also affect the functional state of Th17 lymphocytes. For example, colonization of *Allobaculum bacteria* induces the formation of Th17 lymphocytes in the lamina proper of the small intestine and also increases the severity of neuroinflammation compared to germ-free mice [89]. Monocolonization with another bacterium, *L. reuteri*, did not affect the severity of EAE [90]. Intramuscular Th17 cells are a reservoir of pathogenic Th17lymphocytes. In the mouse EAE model, non-pathogenic Th17intestinal lymphocytes were shown to migrate to the central nervous system via the draining lymph nodes and spleen, where they differentiate into highly pathogenic cells, and trigger neuroinflammation [42]. Mice deficient in IL-17A/F showed a change in the composition of the microbiota and resistance to the development of autoimmune encephalomyelitis [69]. Similar changes were found in antibiotic-treatment mice, which reduced the number of Th17 lymphocytes in the periphery [42,91]. Restoration of the microbiota or administration of IL-17A increases the sensitivity of animals to the development of neuroinflammation [69]. Thus, the microbiota plays a significant role in the differentiation and functional maturation of Th17 lymphocytes. The above data allow us to conclude that Th17 lymphocytes may be the pathogenetic link in gut–brain-axis signaling, which increases the clinical symptoms of depression due to impaired BBB permeability and infiltration by pathogenic Th17 lymphocytes, triggering neuroinflammation. 

## 3. For and against Th17 Lymphocyte Involvement in the Pathogenesis of Depression

In the literature, there is an increasing amount of data confirming the possible role of Th17 lymphocytes and IL-17A in the pathogenesis of depression [2,92,93,94,95,96,97,98,99,100,101,102,103,104,105,106,107]. In the peripheral blood of depressed patients, the level of Th17 cells and IL-17a is significantly increased, the level of regulatory T cells is reduced, and the level of RORγT mRNA in peripheral blood lymphocytes is higher [92,94,95,97]. In patients with treatment-resistant depression, higher levels of IL-17A were found in the blood [3] (Nothdurfter et al., 2021), and pre-treatment level of RORyt mRNA can serve as a biomarker of response to therapy [108]. In patients with a high risk of suicide, a robust increase in the number of Th17 lymphocytes in the blood was observed [106]. In children and adolescents with obsessive-compulsive disorder, an increase in Th17 lymphocytes and IL-17A was found, but their level did not always correlate with the duration and severity of obsessive-compulsive disorder symptoms [107,109]. In pregnant women, starting from the second trimester, with severe symptoms of anxiety and/or depression, an increased level of Th17 cytokines (IL-17A and IL-22) was observed in the blood, which positively correlates with the score of the Hamilton Depression Rating Scale [101], and correlate with the elevated risk of postpartum depression and anxiety [102]. In allergic and autoimmune diseases, a correlation has been shown between patients’ anxiety levels and IL-17A [96,103,110]. In the blood lymphocytes of depressed patients, the level of mRNA IL-17 is increased [2], which is associated with the severity of anhedonia symptoms [99]. Major depressive disorder (MDD) patients showed abnormal circulating CD4+ T lymphocytes with expansion of the IL-17 and TNF-alpha expressing cells as well as increased levels of peripheral IL-17 and TNF-alpha [92]. Ketamine, which has successfully passed phase 2 clinical trials as a candidate for the treatment of depression [111], is able to reduce the differentiation and proliferation of Th17 lymphocytes [112]. Carvalho Alves, 2020, showed that melancholic features such as depressed mood, psychic anxiety, and guilty feelings correlate with the anti-inflammatory cytokine profile (higher IL-4 levels, reduced IL-17 and IL-2, respectively). At the same time, in depressed patients, IL-2, IL-6, IL-10, and IL-17 levels were higher than those of controls [104]. However, there is evidence that does not support an association between plasma IL-17A, depression, and response to therapy [113,114,115]. No differences were found in plasma IL-23 and IL-17 levels in depressed patients and conditionally healthy donors [114,115], and therapy for 6 weeks with various antidepressants did not change baseline cytokine levels [114], although this may be due to the small sample size, gender, and non-correct survey of conditionally healthy donors. Interestingly, in late-life depression, IL-17A levels do not correlate with the severity of depressive symptoms but correlate with cognitive assessments [113]. All this further highlights the complexity of the pathophysiology of depression, the involvement of IL-17 and, indirectly, Th17 lymphocytes in the development of symptoms of depression, and the need for further research.

In animal models, there is also evidence indicating the role of inflammation and Th17 lymphocytes in the development of anxiety and depression-like behavior in models of acute and chronic stress [70,81,116,117,118]. These types of chronic stress (cumulative stress, restrain stress, unpredictable stress) lead to activation of microglia in the animal brain [70,116], impaired BBB permeability [70], and neuroinflammation [15,119]. In the peripheral blood and brain tissues of stressed animals, there is an increase in the production of TNF, IL-6, and IL-17A, as well as the number of Th17 lymphocytes [25,116,117]. Administration of the cytokine IL-17 directly induces depressive-like behavior in mice [117] through activation of the NF-βkB and p38MAPK pathways [120]. Attenuation of neuronal transmission of IL-17A signals (genetic deletion IL-17a receptor) reduces the level of anxious behavior in mice [121]. Mice deficient in the RORyt transcription factor did not develop depressive behavior when using the learned helplessness and chronic restraint stress models [116,117]. On the other hand, some authors indicate a decrease in Th17 lymphocyte levels, plasma IL-17, and RORyt levels in the hippocampus after chronic unpredictable mild stress [122].

**Figure 1:**
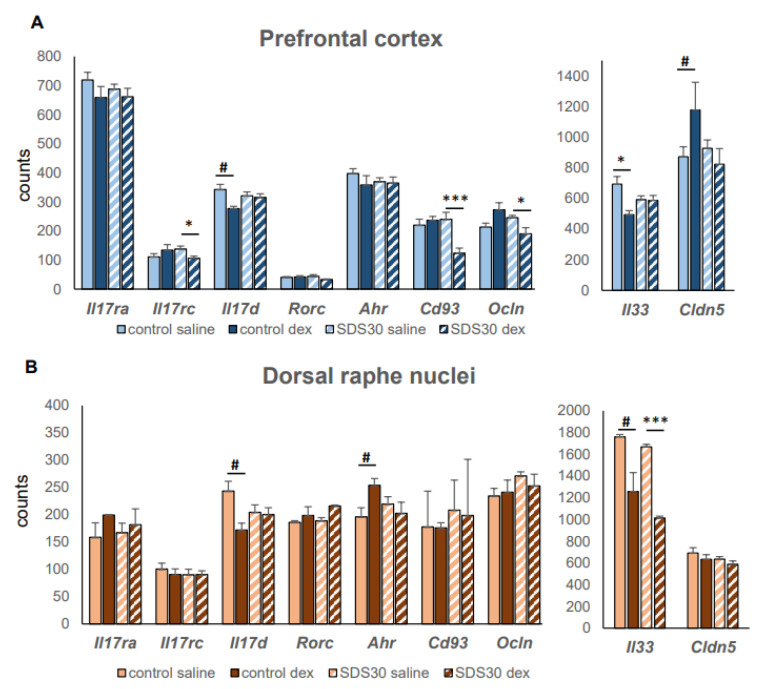
Changes in the level of gene expression in the prefrontal cortex (**A**) and dorsal raphe nuclei (**B**) in control and depressive (30 d social defeat stress, SDS30) mice. The level of gene expression was taken from our previous transcriptome analysis (NCBI: BioProject: PRJNA846517). Control C57Bl/6 male mice and mice with 30 days of experience of social defeat stress were treatment with dexamethasone (2 mg/kg, ip); after 6 h the mice were removed from the experiment, RNA was isolated and mRNA libraries were prepared. *—adjusted *p* < 0.05, ***—adjusted *p* < 0.001- compared with dexamethasone treatment in each group with correction for multiple comparisons; #—*p* <0.05 compared with dexamethasone treatment in each group without correction for multiple comparisons.

In our recent study of the prefrontal cortex (PFC) and dorsal raphe nuclei (DRN) transcriptome after 30-day social defeat stress (BioProject: PRJNA846517), which leads to the development of a depressive state in mice [123]. After 30 days of experience with social defeat, mice were treatment with dexamethasone (ip, 2 mg/kg), and 6 h later the mice were euthanized. We isolated RNA from the PFC and DNS and analyzed the level of gene expression using RNA-seq. We showed that chronic stress alters the sensitivity of gene expression in these structures to dexamethasone, and is more pronounced in glial cells (astrocytes, microglia) and endothelial cells, which confirms the formation of GC resistance in depression (article in preparation). We found a change in the GC sensitivity of individual genes, which indicates the possible involvement of Th17-related pathways in those observed under stress (Figure 1). 

An analysis of our data, as well as a meta-analysis of PFC transcriptomes from other social defeat stress experiments [124], showed that PFC and DRN only the expression of the *Il17d* gene encoding the corresponding cytokine was significantly detected (Figure 1). IL-17D is the least studied cytokine from the IL-17 family. It has been shown that in the periphery, it participates in the regulation of intestinal homeostasis by interacting with ILC3 (innate counterparts of T-helper 17 cells) cells via the non-canonical CD93 receptor, the deficiency of which reduces the differentiation of ILC3 cells and the production of IL-22, necessary for protection against autoimmune colitis [125]. In our study, the development of a depressive state in mice led to a decrease in the sensitivity of Il17d expression to dexamethasone in both brain structures (Figure 1) and in the prefrontal cortex, dexamethasone significantly reduces *Cd93* expression in depressed animals, which suggests a possible decrease in the activity of resident ILC3s brain cells. On the other hand, CD93 expression is also characteristic of endothelial cells and microglia. However, it is not known whether there is a similar mechanism of interaction of IL-17D via CD93 with brain cells. 

Among the IL-17 family signaling pathway genes in PFC and DRN, we detected the expression of two IL-17 receptor subunits, *il17ra*, and *Il17rc*. The expression of the *il17ra* subunit is insensitive to dexamethasone in both depressed and control mice, while *Il17rc* expression responds to dexamethasone only in the PFC of depressed animals. IL-17RA has been observed at high levels in various tissues, binds with high affinity to IL-17A, and mediates the main pro-inflammatory effects of this cytokine. The insensitivity of its expression level to GC may explain the GC resistance of Th17-induced inflammation. IL-17RC is part of the IL-17R receptor complex consisting of IL-17RA and IL-17RC. For peripheral inflammation, it has been shown that disruption of the expression and activation of the IL-17RA/IL-17RC complex on Th17 cells increases IL-17A production and leads to an accelerated course of experimental sickle-shaped glomerulonephritis, psoriasis, and colitis [126]. Thus, treatment of depressed animals with high doses of dexamethasone by reducing the level of IL-17RC can lead to a disruption in the formation of the IL-17RA/IL-17RC complex and an increase in IL-17A production, increasing Th17-mediated inflammation in the brain. On the other hand, total IL-17RC knockout did not affect the course of experimental sickle-shaped glomerulonephritis, and knockdown *Il17rc* led to a milder form of autoimmune encephalomyelitis [127], which contradicts the hypothesis of increased inflammation with a decrease in IL-17RC levels. 

In adipose tissue, loss of IL-17RC expression is accompanied by a decrease in TGF-β1 expression [128]. A decrease in TGF-β1 in brain tissues may be accompanied by increased neuronal death and microgliosis [129]. IL-17RC has been shown to play a different role as a negative regulator in the control of homeostatic proliferation and stress-induced apoptosis in tumor cell lines [130].

In addition to the main participants of the Th17 pathway (Il17d, Il17ra, and Il17rc), the developing depressive state in mice is accompanied by changes in GC sensitivity and a number of other genes that indirectly reflect the effect of Th17 lymphocyte activation. Thus, an increase in the sensitivity of expression of *Il33* in DRN, a cytokine that is involved in maintaining the functional state of pathogenic Th17 lymphocytes, promotes a reduced expression of pro-inflammatory genes, including *Tbx21*, *Ifng*, and *Csf2*, and an increase in *Il10*, an anti-inflammatory cytokine [131]. Dexamethasone has been shown to reduce the expression of *Ocln* and *Cldn5 mRNA* in PFC, although normally GC increases the level of junctional proteins (occludin, claudin-5, tjp11, and VE-cadherin) [132]. This decrease in tight protein expression may be related to the effect of IL-17A on endothelial cells described above. 

Unfortunately, the transcriptome analysis performed does not allow us to determine the specific cell types expressing the described markers. This indicates the need for further research involving single-cell technologies to determine the contribution of Th-17 lymphocytes and IL-17 signaling pathways to the development of depressive behavior under the influence of chronic social stress. 

Thus, the accumulated data does not refute the involvement of Th17 lymphocytes and IL-17 in the pathophysiology of depression, but a clear understanding of their mechanisms of action on the course of the disease has not yet been obtained. It is still not fully understood exactly how Th17 lymphocytes are involved and/or trigger neuroinflammation in depression, how cross-talk between Th17 cells and glial and endothelial cells occurs, and what effect the microbiota has on the inflammatory status of Th17 lymphocytes. 

### Effect of Peripheral Biogenic Amines on Th17 Lymphocyte Differentiation 

In addition to affecting the Th17-type immune response, chronic unpredictable mild stress leads to a significant increase in the level of serotonin (5-HT) and tryptophan hydroxylase 1 (Tph1)—5-HT synthesis enzyme in the colon, but a decrease in them in the prefrontal cortex and hippocampus [25]. Serotonin is one of the neurotransmitters of the central nervous system and is involved in the regulation of mood, appetite, sleep, and metabolism. As noted in the introduction, the serotonin theory still remains dominant in the pathogenesis of depression, and the response to therapy is associated with higher baseline serotonin levels in plasma [133,134,135], and a decrease in blood serotonin level during treatment was associated with a better response to therapy. In vitro studies have shown that serotonin attenuates the proliferation and production of IFN-y and IL-17, but increases IL-10 production in activated T-cell cultures [103,105] and may have an immunomodulatory effect on CD4+ T-cell differentiation towards regulatory populations [136]. Fluoxetine, a selective serotonin reuptake inhibitor, suppresses the production of IL-17, IFN-g, and GM-CSF, but not IL-21, by activated CD4+ T cells in vitro, and blockade of 5-HT2B-receptors with specific antagonist reduced the inhibitory effect of fluoxetine on IL-17, IFN-γ, and GM-CSF production in MS patients [137]. Serotonin receptors 5-HT1aR and 5-HT2R were expressed by the CD4+ T-cell subsets [136] 5-HTR1a was expressed at a higher level in naïve T cells compared to effector T cells and Treg cells, while 5-HTR2 had significantly higher expression in Treg. In depressed patients, there is a decrease in 5-1HT1aR expression on PBMC, especially on CD4+CD4+CD25+ Treg cells [138]. In addition, Treg has been shown to express tryptophan hydroxylase, which converts tryptophan to serotonin, while Th17 lymphocytes express a large amount of the enzyme IDO1, which is involved in the conversion of tryptophate to kynurenine [136]. 

Based on the above data, it can be expected that a decrease in serotonin levels in depression leads to easier activation of Th17 lymphocytes and increased production of pro-inflammatory cytokines. The use of serotonin-type antidepressants, along with increasing serotonin levels, also reduces Th17-induced inflammation.

In turn, pro-inflammatory cytokines affect serotonin metabolism [139]. In response to pro-inflammatory cytokines, expression of the enzyme indolamine-2,3-dioxygenase (IDO) increases in various cells of the body, including microglia and astrocytes, which catalyzes the limiting stage of catabolism of the essential amino acid tryptophan (Trp) via the kynurenine pathway, reducing the availability of tryptophan for serotonin synthesis and increasing the formation of kynurenine (Kyn) and related neuroactive metabolites of the kynurenine pathway, neuroprotective kynurenic acid (KynA) or 3-hydroxykynurenine (3HK), and then neurotoxic quinolinic acid (QuinA) [134,140]. Quinolinic acid is an agonist of the N-methyl—d-aspartate (NMDA) receptor and promotes glutamate accumulation in the synaptic cleft by increasing neurotransmitter release and reducing its reuptake [141]. Plasma tryptophan and kynurenine levels have been shown to be reduced in depression [142] and negatively correlated to the severity of MDD symptoms [140,143] and with suicidal thoughts [144,145]. In turn, kynurenine inhibits the activity of RORyt, which promotes the differentiation of pro-inflammatory Th17 cells [146], so a decrease in kynurenine can contribute to the differentiation of pathogenic Th17 cells in the periphery. The majority of plasma KYN is synthesized in the liver with liver-specific tryptophan 2,3-Dioxygenase (TDO2), and peripheral KYN is the source of ~60% of KYN in the brain [141]. KYN is also an endogenous ligand of the transcription factor AHR (aryl hydrocarbon receptor) [147]. In mice, activation of the transcription factor AHR has been shown to be necessary for the differentiation of non-pathogenic conventional Th17 lymphocytes and FoxP3-Tregs but does not affect the differentiation of IL-23-induced pathogenic Th17 cells [148,149]. In humans, the role of AHR in Th17 lymphocyte differentiation is ambiguous; depending on the microenvironment conditions and ligand, activation of AHR can both promote and inhibit Th17 differentiation [148]. If we assume that in humans, KYN has a similar effect on the differentiation of Th17 and Treg as in mice, then a decrease in KYN levels, in addition to directly affecting RORyt activity, in depression will reduce the differentiation of non-pathogenic Th17 and regulatory T cells through exposure to AHR activity. In depressive patients with suicidal thoughts, the Kyn/Trp ratio has been shown to be positively associated with both cortisol and pro-inflammatory cytokine levels [145].

Dopamine (DA) is another important neurotransmitter, a decrease in the level of which in the brain is associated with the development of anhedonia, the most severe symptom of depression. In the periphery, DA is associated with homeostatic regulation of blood pressure, respiration, gastrointestinal motility, insulin production, circadian rhythms, and immune response [150]. Peripheral dopamine is also strongly associated with the HPA axis and stress response, with higher stress levels associated with increased dopamine release [150]. Some patients with mild anxiety and depression, especially women with compulsive binge eating, were found to have increased blood dopamine levels [151,152,153]. DA amplifies glucocorticoid-resistant Th17 phenotype in MS patients, and this phenomenon could be, at least in part, due to its ability to induce IL-6 production by monocytes and CD4+ T cells [154]. 

It is important to remember that plasma levels of 5-HT and DA may not reflect the levels of neurotransmitters in the brain, and, therefore, cannot be used as a reliable indicator of altered mood and behavior states. It is also known that both peripheral and central serotonin and dopamine do not cross the blood–brain barrier. Given current research and, consequently, the increasing understanding of the complexity of the pathophysiology of depression (for example, the cytokine depression hypothesis, the brain–gut–microbiome axis depression hypothesis), it is still not clear whether the manifestation of peripheral inflammation is a cause or a consequence of major depression. However, due to the differentiation of lymphocytes in the periphery, the level of neurotransmitters in plasma may be important for determining the functional state of immune cells migrating to brain tissues. The above data suggest that a reduced level of 5-HT and an increased level of DA in the blood of patients with depression contribute to the differentiation of pathogenic Th17-lymphocytes resistant to the action of glucocorticoids. Unfortunately, when preparing the review, we were unable to find studies that would assess the level of Th17-type immune response in depressed patients with demonstrated glucocorticoid resistance. Such a study will allow us to verify this data and discover new mechanisms and possible targets in this group of patients. 

## 4. Glucocorticoid Resistance and Th17 Lymphocytes

Glucocorticoids (GCs) have an immunosuppressive effect on almost all types of immune cells, regulating the phenotype, survival, and cell functions of the innate and adaptive immune response. GCs exhibit anti-apoptotic effects, promoting the survival of anti-inflammatory cells, and also inhibit the immunostimulating functions of pro-inflammatory effector cells, inhibiting the release of various pro-inflammatory mediators, such as cytokines, chemokines, and active oxygen, through various mechanisms. In lymphocytes, GC inhibits the synthesis of Th1 cytokines, such as IL-2 and IFN-y, and reduces the activity and expression of Th2 cytokines (IL-4) [155]. However, unlike Th1 and Th2 cells that are sensitive to glucocorticoid suppression, pathogenic inflammatory Th17 lymphocytes are resistant to glucocorticoid suppression [4,7,8,9]. Moreover, the addition of dexamethasone to a culture of proliferating CD4+ T cells under Th-17 polarizing conditions (with TGF-β, IL-6, and IL-23) strongly enhanced Th17 differentiation [156]. When using gene-expression profiling, it was shown that human Th17 lymphocytes, regardless of the general sensitivity of the donor to GCs, exhibit restricted genome-wide responses to glucocorticoids in vitro and that this is independent of glucocorticoid receptor translocation or isoform expression [8], but have sensitivity to the inhibitory effect of cyclosporin A, a powerful immunosuppressant. The authors conclude that the effectiveness of cyclosporine A in the treatment of steroid resistance may be due to its selective inhibition of Th17 cells [8]. Infiltration of lung tissue by Th17 lymphocytes and their production of cytokines in a mouse model of severe asthma was enhanced by GCs [9], which confirms the role of Th17 cells in GC-resistant inflammatory conditions such as asthma [156]. In difficult-to-treat asthma in humans, an increase in Th17 cytokines causes the upregulation of glucocorticoid receptor-beta (GR-β) [157,158], contributing to the dysregulation of the GRα/GRβ ratio [159] and disrupting GC binding to GR and its translocation to the nucleus. 

Among the possible reasons for the formation of such resistance of Th17 lymphocytes to GC, a number of factors can be distinguished. One of the factors protecting Th17 cells from GC-induced apoptosis is the high expression of Bcl-2. Knockdown of Bcl-2 sensitized Th17 cells to dexamethasone-induced apoptosis in both mice and humans [7]. In addition, RORyt, the main differentiation factor of Th17, can also counteract GC-induced apoptosis by inducing the expression of BCL-2 family members Bcl-xL and Bcl-2 [160,161]. Stat3, the second most important transcription factor in Th17 cells, can counteract the suppressive effect of GC by competitively binding to the same promoters as GR (recruitment of GR to DNA-bound Stat3 is associated with trans-repression or transcriptional antagonism) [162]. Moreover, STAT3 phosphorylation in Th17 cells was insensitive to glucocorticoid inhibition [7]. This Stat3 status means that the production of IL-17A, but not IL-22 or GM-CSF, from pathogenic Th17 cells is not sensitive to the action of glucocorticoids [7]. Features of Th17 lymphocyte metabolism also ensure their resistance to the inhibitory effect of GC: Th17 cells have a higher level of glucose and glutaminolysis metabolism than other GC-sensitive T-helper cell subpopulations [163]. Dexamethasone has been shown to increase the activity of glutaminase (GLS) [164] (converts glutamine to glutamate), which directs the activation of T cells towards Th17, while simultaneously disrupting Th1 differentiation and their effector function [165].

IFN I type, an increase in which is also found in depression, can induce GC resistance in interferopathies through reduced expression of glucocorticoid-induced leucine zipper protein (GILZ) (TSC22D3 gene) [166]. The effect of IFN on GILZ expression was dependent on the JAK1/Tyk2 pathway, as indicated by the loss of the inhibitory effect of IFN on GILZ in the presence of JAK inhibitors [166]. In the damaged skin of psoriasis patients, low levels of GILZ were found, which is inversely correlated with the levels of IL-23, IL-17A, IL-22, and STAT3, key factors of Th17 lymphocytes, indicating the existence of a regulatory loop between GILZ and Th17 activity [43]. It is assumed that GILZ is a negative regulator of Th17 differentiation, acting through direct binding to the promoter regions of key Th17 genes, competing with the Th17-positive regulators BATF, STAT3, IRF4, and RORyt for binding sites [5]. In addition, long-term exposure to high concentrations of IL-6, also characteristic of depression [20,21], increases the production of IL-17 by Th17 lymphocytes and additionally reduces corticoid inhibitory effects on activated T cells [167], and the use of anti-IL-6R restored the ability of hydrocortisone to inhibit both T-cell proliferation and IL-17 production. 

By itself, IL-17A inhibits the expression of canonical corticosteroid genes, such as HSD11B2 and FKBP5 [168]. Dexamethasone does not prevent the secretion of IL-17A-induced pro-inflammatory cytokines (TNF, IL-1b, IL-6) by primary human airway epithelial cells, inducing specific cell insensitivity to dexamethasone and the development of steroid-resistant neutrophilic airway inflammation [168,169], and in some cases, even increasing IL-17A production [170] and exacerbating inflammation [171]. Stimulation of epithelial cells by IL-17A leads to a shift towards the pro-inflammatory phenotype, which is also not prevented by dexamethasone [172,173]. Treatment of fibroblasts and epithelial cells with other Th17-regulatory cytokines (IL-22, IL-21, IL-23) effectively inhibits DEX-induced apoptosis, probably due to increased phosphorylation of STAT3 [174]. IL-17A receptors are also known to transmit their signals via p38-MAPK, an extracellular signaling kinase, and phosphoinositide-3 kinases (PI3K), which inhibit GR function by acting on GR translocation or GR-mediated gene transcription [23,172,175]. IL-17A causes a decrease in the activity of histone deacetylase 2 (HDAC2), and overexpression of HDAC2 can restore IL-17A-induced glucocorticoid insensitivity [172]. It is worth noting that the GC sensitivity of other cell populations is poorly understood.

Despite these data, glucocorticoid therapy is still successfully used to treat Th-dependent autoimmune diseases: psoriasis [176], multiple sclerosis [177], and bronchial asthma [178]. Even healthy donors’ lymphocytes are known to have different sensitivities to glucocorticoids [13]. It is possible that there are separate subgroups of Th17 lymphocytes with different sensitivity to GCs. A subtype of pathogenic Th17 lymphocytes, stably express P-glycoprotein (P-gp)/multi-drug resistance type 1 (MDR1), has been shown to produce IL-17A, IL-17F, IL-22, and IFNγ simultaneously and is resistant to GC [179]. GC has been shown to increase the level of P-glycoprotein (P-gp)/multi-drug resistance type 1 (MDR1) in brain endothelial cells [180] and in lymphocytes of patients with rheumatoid arthritis [179], which may lead to drug resistance in patients with autoimmune diseases [179,181]. Under the influence of glucocorticoids, the number of MDR+Th17 lymphocytes resistant to GC increases in mixed cultures of lymphocytes, but blocking MDR1 in Th17 cells does not increase sensitivity to GCs [182]. It can be assumed that long-term GC exposure leads to apoptosis of T cells sensitive to their action, and the remaining resistant populations will exacerbate the existing disease. 

Steroid resistance is a hallmark of depression. It is not known whether Th17 lymphocytes and IL-17A have the same effect on glial cells as on bronchial epithelial cells and lymphocytes. However, in multiple sclerosis, GC resistance is accompanied by the involvement of highly pathogenic Th17 lymphocytes in the central nervous system [183], thereby suggesting the existence of a similar mechanism for glial cells. It is important to emphasize that all these data relate to the use of synthetic glucocorticoids, the question of whether endogenous GCs have similar effects on the cells of the immune system remains open.

## 5. Conclusion, Prospects, and Available Statistical Data Linking SDS and Th17

The data available emphasize the complexity of the pathophysiology of depression, and none of the accepted theories about the occurrence of depression provide a definitive answer to the root cause of the disease. In studying the pathogenesis of depression, opposite mechanisms are very often discussed, for example, high levels of cortisol and pro-inflammatory markers. In our review, we proposed pathogenetic pathways that could explain the coexistence of two mutually exclusive conditions. Unfortunately, there are not many studies on the role of neuroinflammation in the pathogenesis of depression, much less on the role of Th17 lymphocytes, and the amount of information available is limited. The insensitivity of Th17 lymphocytes to glucocorticoids in other pathological conditions unrelated to the central nervous system [8,40] and their associated ability to preserve pathogenetic pro-inflammatory effects on surrounding tissues with increased levels of glucocorticoids suggests their role in chronic neuroinflammation in depressive disorders, especially since confirmation of the existence of Th17 lymphocytes in the brain was found [183]. The high plasticity of Th17 lymphocytes, their ability, depending on the microenvironment conditions, to change their functional properties, become pathogenic or homeostatic, as well as acquire signs of other Th cell subpopulations, is studied in various immunopathogenic conditions, but is practically not studied in depression. The available reviews on the involvement of Th17 lymphocytes in the pathogenesis of depression [98,112] do not take into account either the role of glucocorticoid resistance, which is typical for some patients with depression, or GC resistance of Th17 cells themselves.

The analysis of the effect of glucocorticoid resistance and Th17 lymphocytes on the pathogenesis of depression has a number of limitations regarding the interpretation of data. Firstly, there is the heterogeneity of the experimental data used for analysis (for example, different objects of study—mice or humans, in vivo or in vitro experiments, the type of pathology under study). The heterogeneous design of experiments means that we can use only the most pronounced changes as arguments, but we cannot exclude the influence of less pronounced effects of a particular model. Finally, the third limitation is the morphofunctional differences in the brain between rodents and humans, which in some cases leads to species-specific reactions. Despite the limitations, our analysis indicates the existence of a link between glucocorticoid resistance, Th17 lymphocytes, and the possible development of depression. This finding also confirms the similarity of the changes found in animal models of depression at the cellular and molecular levels with the pathological changes that occur during depression.

In our review, we tried to analyze data that would link glucocorticoid resistance, Th17 lymphocytes, and depressive disorder. It is obvious that glucocorticoid resistance is not a unique condition for one of the body’s systems, but can simultaneously affect different organs and tissues. Thus, these literature data support the concept that Th17 cell-mediated diseases may have signs of glucocorticoid resistance. A number of changes in gene expression, which we have revealed, indicate the formation of glucocorticoid resistance and possible involvement of Th17 lymphocytes in the pathogenesis of a depressive state in mice exposed to chronic social stress. However, further studies are needed to combine these two conditions and investigate the role of glucocorticoid sensitivity in the differentiation and functional state of Th17 cells. Another intriguing question is whether depression, like asthma, can be differentiated into subtypes based on Th17 lymphocyte subpopulations and their sensitivity to glucocorticoids. It is necessary to identify Th17 regulatory factors that will overcome glucocorticoid resistance and improve patients’ response to depression therapy.

## Figures and Tables

**Table 1:** Main features of Th17 lymphocytes.

	Non-Pathogenic Th17	Pathogenic Th17
Factor of differentiation	TGF-β, IL-6	TGF-β, IL-6, IL-23 + IL-1β, IL-12, TNF
Transcription factors	RORγt, STAT3, c-Maf, AHR, Ets1, Batch2, Fosl2, Rbpj	RORγt, STAT3, Atf3, Bhlhe40, Fos, Nr4a1, Eomes, Tbx21
Surface receptors	CCR4, CCR6, IL6R, CD5L	IL-23R, CCR4, CCR6, IL6R, CXCR3
Cytokines	IL-10, IL-9, IL-17, IL-21	IL-17, IL-21, IL-22, GM-CSF, IFNγ, Granzime B
Metabolism	Oxidative phosphorylation	Glycolysis
Function	homeostasis the host defense against fungal and bacterial pathogens in mucosal and epithelial barriers neutrophil recruitment immune-suppressive properties in the gut	inflammation autoimmune disorders, especially multiple sclerosis, psoriasis and rheumatoid arthritis chronic fungi infection, especially Candida

Abbreviations: AHR—aryl hydrocarbon receptor; CCR—C-C-chemokine receptor; Eomes—eomesodermin; GM-CSF—granulocyte-monocyte colony-stimulating factor; IFN—interferon; IL—interleukin; RORγt—RAR-related orphan receptor γ; TGF-β—transforming growth factor beta; STAT—signal transducer and activator of transcription; Tbx21—T-cell-specific T-box transcription factor; TNF—tumor necrosis factor.

## Data Availability

BioProject: PRJNA846517.

## Abbreviations

5-HT	5-hydroxytryptamine, serotonin
ACTH	adrenocorticotropin
AHR	aryl hydrocarbon receptor
BBB	blood–brain barrier
CNS	central nervous system
CRH	corticotropin-releasing hormone
DA	dopamine
EAE	experimental autoimmune encephalomyelitis
GM-CSF	granulocyte-monocyte colony-stimulating factor
GR	glucocorticoid receptor
GS	glucocorticoid
HPA	hypothalamic-pituitary-adrenal axis
IDO	indolamine-2,3-dioxygenase
IFN	interferon
IL	interleukin
MDD	major depression disorders
MS	multiple sclerosis
PBMC	peripheral blood mononuclear cells
RORγt	RAR-related orphan receptor γ
TGF-β	transforming growth factor beta
Th	T-helper cells
TNF	tumor necrosis factor
Tph1	tryptophan hydroxylase 1
Tregs	regulatory T-helper cells
